# Multivessel myocardial bridging in a patient with spiral hypertrophic cardiomyopathy

**DOI:** 10.21542/gcsp.2016.30

**Published:** 2016-09-30

**Authors:** Timothy Fritz, Wissam Abdallah, Richard McNamara

**Affiliations:** Spectrum Health Frederik Meijer Heart & Vascular Institute, Grand Rapids, Michigan, USA

## Abstract

Myocardial bridging is commonly observed in hypertrophic cardiomyopathy, usually confined to the left anterior descending (LAD), and correlates to the hypertrophic septum. We present a patient with unique spiral hypertrophic cardiomyopathy (HCM) and compression of all three coronary arteries corresponding to this hypertrophy pattern.

## Introduction

Chest pain is a frequent complaint in 25–50% of patients with HCM. Several mechanisms somewhat unique to HCM have been proposed, including intramural hyperplasia of the coronary artery, severe dynamic outflow tract obstruction, and myocardial bridging. Myocardial bridging occurs when a coronary artery, embedded deep in the myocardium, is compressed during systolic contraction.

The prevalence of myocardial bridging is highly variable depending on the method of evaluation and the patient population. In the general population, bridging is seen on coronary angiography in less than 3% of patients. However, coronary computed tomography and autopsy demonstrate higher rates of bridging in the 25–50% range.

Bridging does appear to be more common in hypertrophic cardiomyopathy, demonstrated in nearly 30% of patients with coronary angiography. Bridging is usually confined to the LAD which corresponds to the marked septal hypertrophy pattern that is most common in HCM. Rarely is multi-vessel bridging observed^1^.

The clinical significance of bridging is controversial^1^. Most bridging occurs in systole and therefore does not alter myocardial perfusion which is primarily in diastole. However, bridging of long segments that supply a large area of myocardium may produce definite diastolic compression and reduction in flow. Fraction flow reserve (FFR) has demonstrated resting and inducible ischemia in myocardial bridging segments^2^.

Spiral hypertrophic cardiomyopathy is a unique subset of HCM with segmental hypertrophy that spirals down the left ventricle in a counterclockwise fashion from base to apex. Hypertrophy begins at the anteroseptal base, descends into the mid and distal inferior septum, and finally terminates in the inferolateral apical segment^3^. Late gadolinium enhancement is reportedly infrequent and adverse clinical outcomes are fewer than other forms of HCM^4^.

We present a patient with spiral hypertrophic cardiomyopathy who demonstrates complete systolic obliteration of all three main coronary arteries corresponding to the spiral hypertrophy demonstrated on magnetic resonance imaging (MRI).

## Patient and methodology

A 40-year-old man presented to the emergency room with progressive chest pain. The chest pressure was central squeezing pressure, occurring with minimal exertion, worse after meals, and particularly worse if he did not take his medications regularly. He was diagnosed with HCM at age 19, was followed by his primary care physician, but had no cardiology evaluation in over 10 years. His treatment consisted of extended release beta blocker and calcium blockers.

**Figure 1.**
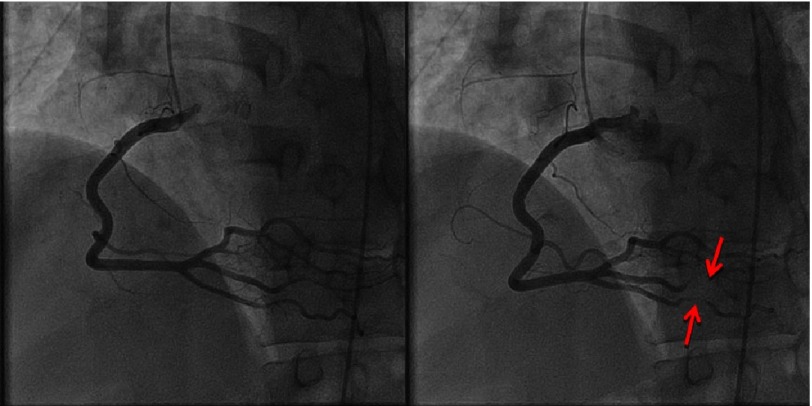
RCA in diastole (A) and systole (B). Myocardial bridging in the distal RCA as noted in systole (B).

**Figure 2.**
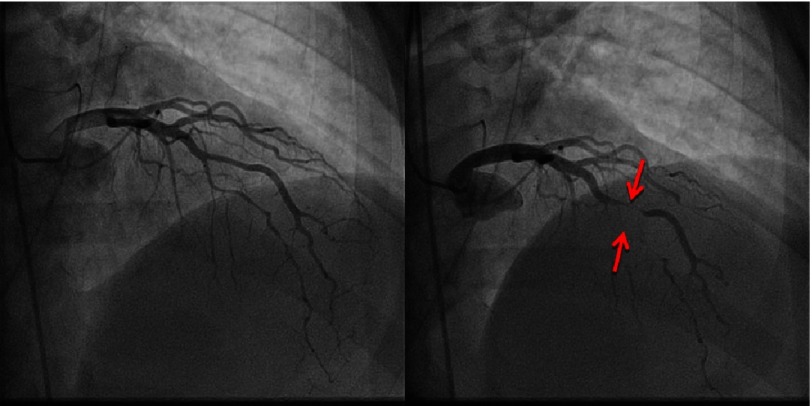
LAD artery in diastole (A) and systole (B). Myocardial bridging in the mid LAD artery and diagonal artery as noted in systole (B).

**Figure 3.**
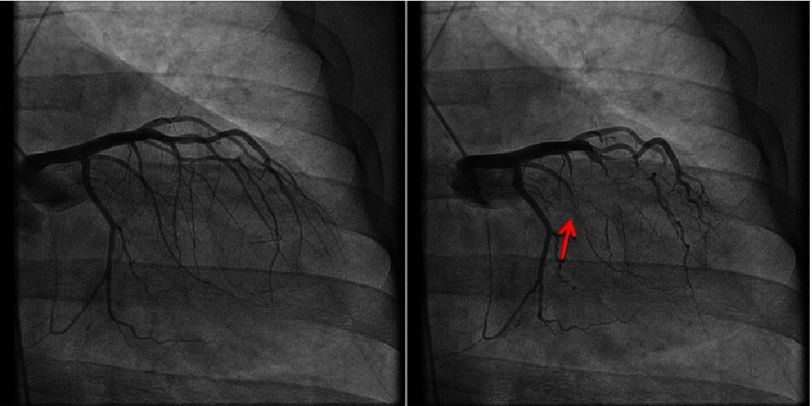
Left circumflex artery in diastole (A) and systole (B). Myocardial bridging in the obtuse marginal branches as noted in systole (B).

**Figure 4.**
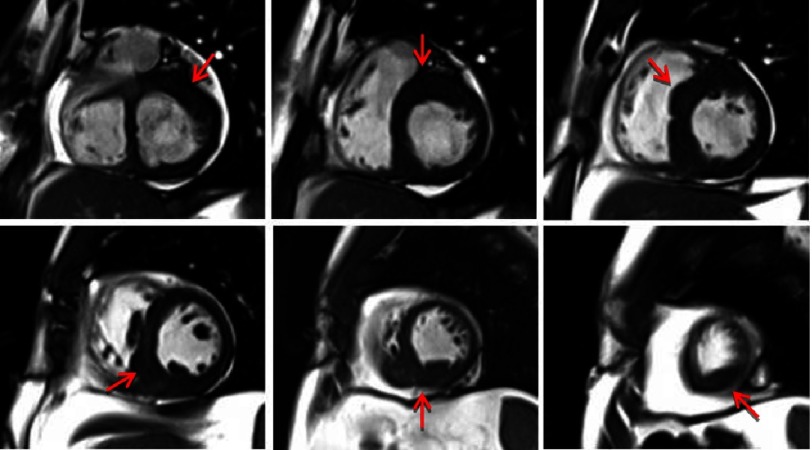
Steady-state free precession images of the left ventricle from base to apex in the short axis orientation showing a spiral hypertrophy pattern extending from the basal anterior wall, basal to mid septal, and mid to distal inferoseptal/inferior wall.

 His EKG demonstrated marked LVH with deep symmetrical T waves across the precordium. During his observation in the ER, a peculiar accelerated idioventricular rhythm, rate of 120 bpm, occurred at the termination of his episodes of chest discomfort. He underwent emergent left heart catheterization with coronary angiography and subsequent MRI imaging.

## Coronary and cardiac MRI imaging

Coronary angiography demonstrated complete obliteration of all 3 coronary distributions during systole.

## Lessons learned

Although myocardial bridging is common in HCM, it is usually confined to the LAD corresponding to the typical pattern of septal hypertrophy. The clinical significance of hypertrophy remains controversial. We suspect the dramatic and extensive bridging in this patient contributed to his disabling chest pain. We present a patient with a peculiar spiral pattern of hypertrophy and bridging of all three coronary arteries corresponding to these hypertrophied segments.

## LIST OF ABBREVIATIONS

HCM: Hypertrophic Cardiomyopathy
CMB: Coronary Myocardial Bridging
MRI: Magnetic Resonance Imaging
LAD: Left Anterior Descending
LVH: Left Ventricular Hypertrophy
